# Separation of stroke from vestibular neuritis using the video head impulse test: machine learning models versus expert clinicians

**DOI:** 10.1007/s00415-025-12918-3

**Published:** 2025-03-05

**Authors:** Chao Wang, Jeevan Sreerama, Benjamin Nham, Nicole Reid, Nese Ozalp, James O. Thomas, Cecilia Cappelen-Smith, Zeljka Calic, Andrew P. Bradshaw, Sally M. Rosengren, Gülden Akdal, G. Michael Halmagyi, Deborah A. Black, David Burke, Mukesh Prasad, Gnana K. Bharathy, Miriam S. Welgampola

**Affiliations:** 1 Central Clinical School, University of Sydney, Sydney, NSW Australia; 2 Institute of Clinical Neurosciences, Royal Prince Alfred Hospital, Sydney, NSW Australia; 3 St George and Sutherland Clinical School, University of New South Wales, Sydney, NSW Australia; 4 Department of Neurophysiology, Liverpool Hospital, Sydney, NSW Australia; 5 South Western Sydney Clinical School, University of New South Wales, Sydney, NSW Australia; 6 Faculty of Medicine and Health, University of Sydney, Sydney, NSW Australia; 7 Department of Neurosciences, Institute of Health Sciences, Dokuz Eylül University, Izmir, Türkiye; 8 Department of Neurology, Faculty of Medicine, Dokuz Eylül University, Izmir, Türkiye; 9 School of Computer Science, Faculty of Engineering and Information Technology, University of Technology Sydney, Sydney, NSW Australia

**Keywords:** Stroke, Vestibular neuritis, Artificial intelligence, Machine learning, Video head impulse test

## Abstract

**Background:**

Acute vestibular syndrome usually represents either vestibular neuritis (VN), an innocuous viral illness, or posterior circulation stroke (PCS), a potentially life-threatening event. The video head impulse test (VHIT) is a quantitative measure of the vestibulo-ocular reflex that can distinguish between these two diagnoses. It can be rapidly performed at the bedside by any trained healthcare professional but requires interpretation by an expert clinician. We developed machine learning models to differentiate between PCS and VN using only the VHIT.

**Methods:**

We trained machine learning classification models using unedited head- and eye-velocity data from acute VHIT performed in an Emergency Room on patients presenting with acute vestibular syndrome and whose final diagnosis was VN or PCS. The models were validated using an independent test dataset collected at a second institution. We compared the performance of the models against expert clinicians as well as a widely used VHIT metric: the gain cutoff value.

**Results:**

The training and test datasets comprised 252 and 49 patients, respectively. In the test dataset, the best machine learning model identified VN with 87.8% (95% CI 77.6%–95.9%) accuracy. Model performance was not significantly different (*p* = 0.56) from that of blinded expert clinicians who achieved 85.7% accuracy (75.5%–93.9%) and was superior (*p* = 0.01) to that of the optimal gain cutoff value (75.5% accuracy (63.8%–85.7%)).

**Conclusion:**

Machine learning models can effectively differentiate PCS from VN using only VHIT data, with comparable accuracy to expert clinicians. They hold promise as a tool to assist Emergency Room clinicians evaluating patients with acute vestibular syndrome.

**Supplementary Information:**

The online version contains supplementary material available at 10.1007/s00415-025-12918-3.

## Introduction

**Fig. 1:**
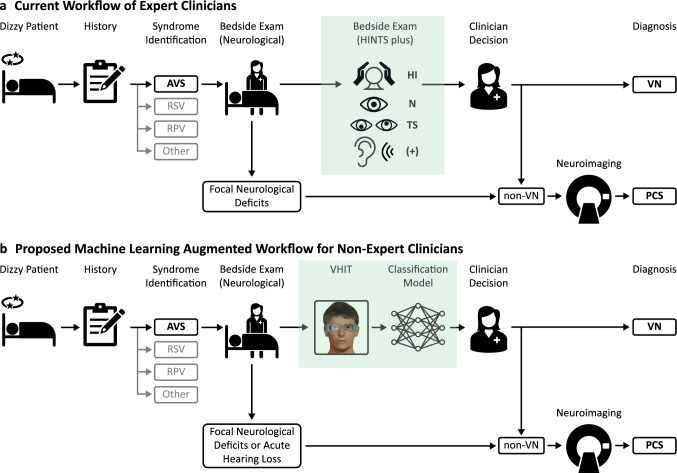
Diagnostic workflow for acute vestibular syndrome. **a** Current workflow used by expert clinicians. **b** Proposed machine learning augmented workflow for non-expert clinicians. The history separates patients with AVS from other vertigo/dizziness syndromes. Currently, expert clinicians can diagnose VN using HINTS plus; non-VN cases receive neuroimaging and are treated as PCS. For non-expert clinicians who are not confident with HINTS, we propose that VHIT with interpretation by a machine learning model would similarly allow accurate diagnosis. Note that the VHIT replaces HINTS and still allows for rapid clinical assessment. *AVS* acute vestibular syndrome, *HINTS* Head Impulse, Nystagmus, Test of Skew, *PCS* posterior circulation stroke, *RPV* recurrent positional vertigo, *RSV* recurrent spontaneous vertigo, *VHIT* video head impulse test, *VN* vestibular neuritis

The acute vestibular syndrome (AVS) refers to sudden onset severe, persistent vertigo and/or imbalance [1] and is a common presentation to the Emergency Room. Correctly identifying the cause is important as it is usually due to one of two conditions with very different therapeutic and prognostic implications: posterior circulation stroke (PCS), which is potentially life-threatening and may necessitate urgent reperfusion therapy, or vestibular neuritis (VN), a relatively benign self-limiting illness. Untrained clinicians may struggle to make the correct diagnosis, especially as focal neurological signs may not be apparent in two-thirds of stroke patients with AVS [2] and so cannot be relied upon. HINTS (Head Impulse, Nystagmus, Test of Skew) is a well-known three-step bedside assessment that, when performed by an expert examiner, can identify stroke in AVS patients with 96% specificity and 100% sensitivity and is more sensitive than early MRI [3]. HINTS became HINTS “plus” [4] by adding bedside screening for new hearing loss as a red flag for ischemia. Figure 1a shows the current clinical workflow of an expert clinician assessing AVS using HINTS. However, the real-life utility of HINTS is limited by a lack of familiarity and confidence in its application among frontline clinicians who assess AVS patients [5]. Non-experts will often perform HINTS incorrectly, particularly the head impulse test [6] which is the most useful component for separating VN and PCS [2]; additionally, they may misinterpret the results even when HINTS is performed correctly, such as by treating a completely normal HINTS as an innocuous finding [7]. Thus, there is need for an alternative to HINTS that can be used by non-expert clinicians.

The video head impulse test (VHIT) [8] is an instrumented, quantitative version of the bedside head impulse test. A full test of all six semicircular canals can be rapidly performed at the bedside in 15 min or less by any trained healthcare professional (such as a doctor, nurse, physiotherapist or audiologist, including Emergency Room staff) and is well-tolerated by symptomatic patients [9]. The VHIT uses specialized goggles to measure a patient’s head and eye position over time in response to a rapid passive head turn. The head and eye traces are used to calculate the vestibulo-ocular reflex (VOR) ‘gain’, a measure of the function of the semicircular canal that is aligned in the same direction as the head turn.

In VN, there is unilateral VOR impairment, which manifests on VHIT as reduced gain with catchup saccades in particular patterns of affected canals [10]. Conversely, posterior circulation stroke patients have variable results on VHIT that range from normal to abnormalities that can either resemble or differ from those seen in VN [11]. In practice, experts can rapidly identify VN on VHIT by its distinctive appearance, enabling its separation from PCS; this is done not by utilizing fixed rules, but by applying pattern recognition-based decision-making with clinical judgment. Previous investigators have used solely an optimal horizontal canal VOR gain cutoff to separate VN and PCS with up to 90% accuracy [12, 13]. However, the optimal gain cutoff value varies widely in different cohorts [14, 15] which limits its generalizability.

Our hypothesis was that the head and eye position data from VHIT testing performed on a widely available commercial system could be exported and used, without additional manual review or processing, to train machine learning models to separate VN and PCS using solely VHIT data with the accuracy of an expert. We compared our models against expert clinicians as well as a simple VOR gain cutoff. To simulate a frontline setting, we used VHIT data that did not undergo post-testing quality control. We demonstrated generalizability of our models by evaluating them using a test dataset collected at a separate institution. Our objective was to develop and evaluate a prediction tool that allows frontline clinicians, without access to neuro-otology expertise, to harness the power of VHIT for more accurate diagnosis of AVS patients. Figure 1b shows a proposed diagnostic workflow for AVS in the Emergency Room that incorporates VHIT together with our classification models as a substitute for HINTS plus.

## Methods

We followed a standard data science lifecycle approach [16, 17] consisting of problem formulation, data acquisition and preparation, model development and evaluation. As a research study culminating in a proof of concept, the last stage of production including deployment, monitoring, and refinement has been excluded from this paper.

### Problem formulation and collaboration

A team of data scientists and neuro-otologists approached the problem of separating vestibular neuritis (VN) from posterior circulation stroke (PCS) using only VHIT data. Our hypothesis was that VHIT data alone might be sufficient to distinguish the two conditions. A panel of clinical experts independently validated the external results. All VHIT data were labeled with the ground truth by diagnosing clinical specialists who used history, examination findings, and results from VHIT, vestibular-evoked myogenic potentials, subjective visual horizontal, audiometry and neuroimaging.

### Participants

VHIT data were collected as separate, consecutively recruited patient cohorts from two major tertiary hospitals with comprehensive stroke services in Sydney, Australia. The first cohort, used for model training, was recruited from Royal Prince Alfred Hospital between March 2018 and March 2023. The second cohort, used for model evaluation, was collected at Liverpool Hospital between May 2018 and September 2021. Both cohorts consisted of patients presenting with sudden onset vertigo/dizziness to the Emergency Room who were referred to the neurology service, had VHIT performed and received a final diagnosis of VN or PCS. This diagnosis acted as the gold standard for comparison against the machine learning models. Patients who presented with dissection already demonstrated on imaging were excluded as VHIT is contraindicated. In the second cohort, patients with focal neurological deficits (namely diplopia, dysarthria, facial/limb weakness or sensory loss and limb ataxia) were also excluded. In both cohorts, VN was diagnosed if patients met all of the following criteria: (1) acute vertigo/dizziness persisting at rest for hours; (2) spontaneous unidirectional nystagmus seen on videonystagmography; (3) either VHIT with impaired VOR gain in a canal pattern consistent with superior, inferior or pan-neuritis, or unilaterally positive bedside head impulse test performed by an experienced examiner plus consistent corresponding catchup saccades on VHIT; and (4) no diffusion restriction on MRI performed > 48 h (performed in first cohort if VHIT suggestive of inferior VN or if skew present, and in second cohort on all patients). Note that our VN criteria included patients with a normal VOR gain on their VHIT provided appropriate catchup saccades were present, consistent with reports in the literature of VN patients showing this result on acute VHIT [11, 18]. All PCS patients in both cohorts had CT and/or MRI imaging confirming ischemic or hemorrhagic stroke. Only patients who were reviewed within 7 days of symptom onset were included.

### Video head impulse testing

The first cohort had VHIT performed by a doctor, nurse, or audiologist, and the second cohort by an audiologist. The ICS impulse system (Natus, CA, USA) was used at both sites. Impulses were collected from all six semicircular canals. Testing usually took about 10–15 min. VHITs were included in this study if they had at least ten impulses recorded from all six semicircular canals (this number of impulses has been suggested as the minimum that allows gain to be accurately estimated without post-processing [19]) with peak head velocity < 250°/s for horizontal canals or < 200°/s for vertical canals. VHITs meeting inclusion criteria did not have manual adjustment or quality review after collection.

### Classification by machine learning models

The VHIT data used for machine learning were extracted directly from the XML files generated by the ICS Impulse software for each VHIT. The VHIT variables in the dataset consist of the head- and eye-velocities recorded over 175 timepoints for each impulse (reflecting the 750 ms data collection duration) as well as identifiers for the corresponding patient, canal, and impulse, and finally which group the patient was in (VN or PCS). Dataset metadata is shown in Supplementary Table 1. Data from the first ten impulses collected per canal per patient were used for model development. See Supplementary Methods for details on how data were prepared for machine learning.

**Fig. 2:**
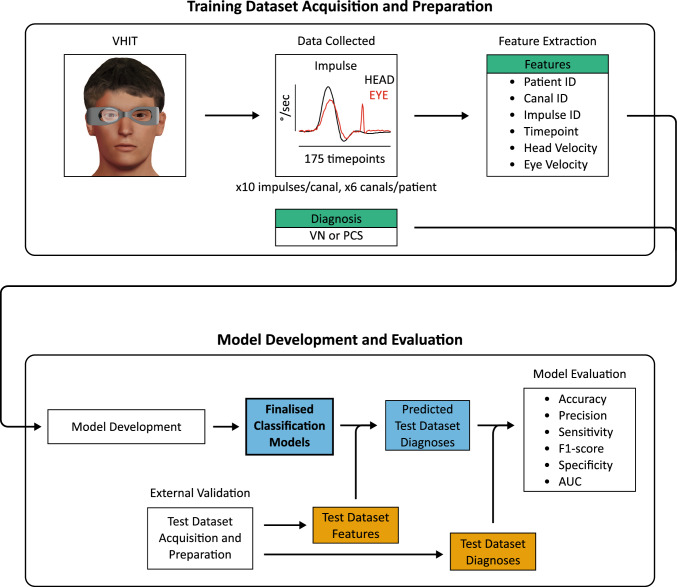
Machine Learning Model Development Workflow. Data from the VHIT was used to train models to classify patients as vestibular neuritis or posterior circulation stroke. Models were developed using data from either the two horizontal semicircular canals or all six canals. The final models were externally validated using a test dataset acquired at a separate institution and prepared in the same manner as the training dataset; the models used the features of the test dataset to predict its diagnoses, which were evaluated against the true diagnoses. *AUC* area under the receiver operating characteristic curve, *PCS* posterior circulation stroke, *VHIT* video head impulse test, *VN* vestibular neuritis

We used the sktime Python machine learning library version 0.28.0 to develop, validate, and evaluate models that classified patients as either VN or PCS based on their VHIT data. For the demographics collected, no group was marginalized or excluded in model development. The training dataset consisted of patients from the first cohort. For comparison, we trained models on either data from all six semicircular canals or data from the two horizontal canals only. Furthermore, as it is not possible to predict which machine learning algorithm would be optimal for a particular dataset, we trialed four different algorithms suited to time-series data: Arsenal [20], catch22 [21], Random Interval Classifier [22] and Rocket [23]. Hyperparameter tuning was performed manually using the training set. The resultant models were then validated by evaluating them on the metrics specified in statistical analysis using an external test set consisting of patients from the second cohort. Figure 2 summarizes the machine learning model development workflow. Finally, we explored the impact on model performance of using different training and test sets derived by combining both cohorts and applying an 80/20 training/test split; again, we compared models generated using the four different algorithms on data from either two or six canals. For evaluation of these models, 5-fold stratified cross-validation was applied, and all models used the same training/test splits.

### Classification by blinded experts

We compared the performance of our models on the test set to the majority opinion of four blinded clinicians with neuro-otology expertise: three neuro-otologists (authors C.W., G.A. and M.W.) and a neuro-otology clinical nurse consultant (author N.R.). In the event of a tie, the result was determined by the opinion of another blinded neuro-otologist (author G.M.H.). The experts had 4–30 years of subspeciality experience; none were affiliated with the hospital at which the test set was recruited. The clinicians independently classified each patient in both the training and test sets as VN or PCS relying solely on a deidentified image of their VHIT showing the traces and gains of the six semicircular canals. This image was adapted from the PDF generated by the ICS Impulse software for each test. The experts were not given criteria to follow, but used their prior experience classifying VN and PCS using the VOR gain, presence and amplitude of catchup saccades, pattern of canal dysfunction (i.e., whether the canal abnormalities were consistent with a superior, inferior or pan-neuritis pattern) and overall waveform of the VHIT response.

### Classification by VOR gain cutoff

We assessed how effectively VN and PCS could be separated using the lower VOR gain (automatically calculated by the ICS Impulse software) from the two horizontal canals. To do this, we evaluated the performance, on both the training and test sets, of a binary classifier that used a cutoff of the optimal VOR gain from the training set as determined by Youden’s index; values below the cutoff were considered VN. Assessing the performance of a VOR gain cutoff in the test set using the optimal result from the training set is comparable to how machine learning models are evaluated on a test set after being developed using a training set.

### Statistical analysis

Statistical analysis was performed using R (version 4.4.0). Statistical significance was defined as *p* ≤ 0.05. Age was compared between the cohorts using the two-tailed independent samples *t* test for PCS and (due to non-normal distribution) the Mann–Whitney U test for VN. Sex was compared between cohorts using the *χ*^2^ test for both diagnoses.

We evaluated the classification methods on both the training and test sets using the metrics (“performance metrics”) of accuracy, precision, sensitivity, F1 score, specificity, and area under the receiver operating characteristic curve (AUC). We derived 95% confidence intervals (CI) using DeLong’s method for AUC [24] and 2000 stratified bootstrap replicates [25] for other variables, except for the models which used an 80/20 split of pooled patients, in which case the five iterations generated by cross-validation were treated as the sample set. As our two classes of VN and PCS had relatively balanced presentation in the training set, we chose accuracy as the primary metric to evaluate performance. However, as the classes were not perfectly balanced, we also considered as an alternative metric the F1 score, which is the harmonic mean of precision and recall (sensitivity). As VHIT is most effective for identifying VN, we defined VN as the positive diagnosis. Performance of the machine learning models was compared against that of the other classification methods using McNemar’s test on the proportion of incorrect predictions [26].

### Sample size calculation

To calculate sample size for separating VN and PCS using unedited VHIT data, we used the first saccade duration as a surrogate VHIT parameter. We conservatively selected first saccade duration out of several VHIT gain and saccade parameters from previous work comparing VN and PCS (Nham, Wang et al. [27]), as its values required the largest sample size to achieve adequate statistical power. Using the reported first saccade duration values, as well as an estimated PCS:VN enrollment ratio of 0.60 (Comolli, Korda et al. [28]), we calculated a sample size of 26 VN and 16 PCS patients to achieve 80% power for separating the two conditions. For both training and test sets, we used all available collected data, and both sets met the sample size requirement.

## Results

**Fig. 3:**
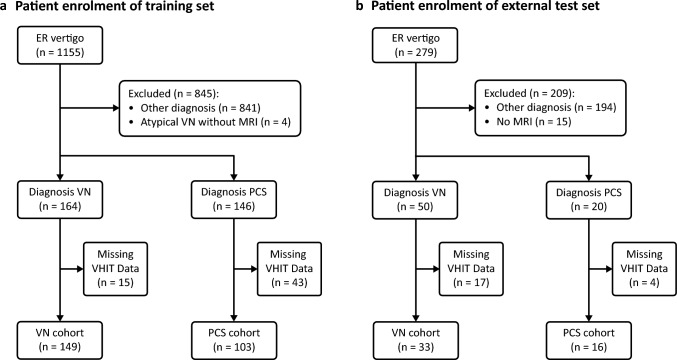
Patient enrolment. **a** Training set. **b** Test set from external institution. Reasons for missing VHIT data for machine learning include unavailable raw data, inability to complete testing of all 6 semicircular canals, inadequate impulses or VHIT contraindicated due to arterial dissection. *ER* emergency room, *VHIT* video head impulse test, *VN* vestibular neuritis, *PCS* posterior circulation stroke

The first cohort consisted of 252 patients (149 with VN and 103 with PCS) and the second cohort had 49 patients (33 with VN and 16 with PCS). Figure 3 shows patient enrollment. There were no statistically significant differences in age or sex distribution between the two cohorts for both VN and PCS. See Supplementary Table 2 for patient characteristics. Overall, patients tolerated VHIT well. During recruitment of the first cohort, VHIT was able to be performed in 100% of VN patients, although 2% did not tolerate vertical canal testing, and 97% of PCS patients (excluding those in which it was contraindicated due to arterial dissection), with 9% unable to complete vertical canal testing. During recruitment of the second cohort, 100% of VN and PCS patients tolerated VHIT (4% of VN patients did not have vertical canal testing).

### Classification by machine learning models

When the first cohort was used as the training set and the second as the test set, classification models using the Rocket algorithm achieved higher accuracies and F1 scores than the other three algorithms, both when trained on data from all six semicircular canals and from just the two horizontal canals. These models detected VN in the test set with 83.7% accuracy (95% CI 73.5%–93.9%) and 87.9% F1 score (95% CI 78.0%–95.5%) using six canals, and with 87.8% accuracy (95% CI 77.6%–95.9%) and 90.9% F1 score (95% CI 82.8%−97.1%) using two canals. All four algorithms performed reasonably well in the test set regardless of whether data from six or two canals were used, with accuracies of 71.4–83.7% and 81.6–87.8%, respectively. Table 1 shows the complete performance metrics of the best six-canal and two-canal machine learning models in both the training and test sets, and Supplementary Table 3 shows the same for models developed using the other algorithms. There was no statistically significant difference between the six- and two-canal models (*p* = 0.32). When the cohorts were pooled and then split 80/20 for training and testing, the best model of those trained on six canals reached 84.7% accuracy (95% CI 79.3%–90.1%) and 87.3% F1 score (95% CI 82.3%–92.2%) in the test set; of the models developed using two canals, the best model achieved 82.7% accuracy (95% CI 77.5%–87.9%) and 85.7% F1 score (95% CI 81.6%–89.7%). The complete performance metrics of all models which used the 80/20 split of pooled patients are shown in Supplementary Table 4.

**Table 1:** Performance metrics of classification methods for separating posterior circulation stroke and vestibular neuritis

	Accuracy, %	Precision, %	Sensitivity, %	F1 score, %	Specificity, %	AUC
Training set						
All canal model	92.5 (88.9–92.5)	94.5 (90.9–97.9)	92.6 (87.9–96.6)	93.6 (90.3–96.2)	92.2 (86.4–97.1)	0.92 (0.89–0.96)
Horizontal canal model	92.9 (89.7–95.6)	95.8 (92.6–98.6)	91.9 (87.3–96.0)	93.8 (90.9–96.5)	94.2 (89.3–98.1)	0.93 (0.90–0.96)
Horizontal canal gain method	84.9 (80.6–94.9)	89.4 (84.8–93.9)	84.6 (78.5–90.0)	86.9 (82.5–91.0)	85.4 (78.6–92.2)	0.85 (0.81–0.89)
Expert clinicians	88.1 (83.7–92.1)	91.0 (86.6–95.0)	88.6 (83.2–93.3)	89.8 (86.0–93.3)	87.4 (80.6–93.2)	0.88 (0.84–0.92)
Test set						
All canal model	83.7 (73.5–93.9)	87.9 (79.5–96.8)	87.9 (75.8–97.0)	87.9 (78.0–95.5)	75.0 (56.3–93.8)	0.81 (0.69–0.94)
Horizontal canal model	87.8 (77.6–95.9)	90.9 (82.9–100)	90.9 (81.8–100)	90.9 (82.8–97.1)	81.3 (62.5–100)	0.86 (0.75–0.97)
Horizontal canal gain method	75.5 (63.3–85.7)	80.0 (71.4–90.0)	84.8 (72.7–97.0)	82.4 (70.8–90.9)	56.3 (31.3–81.3)	0.71 (0.57–0.85)
Expert clinicians	85.7 (75.5–93.9)	90.6 (81.6–100)	87.9 (75.8–97.0)	89.2 (80.6–95.9)	81.3 (62.5–100)	0.85 (0.73–0.96)

The classification methods shown are, in order, the best-performing machine learning model that used data from all six semicircular canals, the best model that only used the two horizontal canals, a binary classifier using the optimal horizontal canal gain cutoff determined by Youden’s index from the training set, and the majority opinion of expert clinicians. Vestibular neuritis was defined as the positive class. The brackets indicate 95% CI. F1 score is the harmonic mean of precision and recall (sensitivity)

*AUC* area under the receiver operating characteristic curve

### Classification by blinded experts

The majority opinion of the blinded experts using data from all six canals identified VN in the second patient cohort (the test set) with 85.7% accuracy (95% CI 75.5%–93.9%) and 89.2% F1 score (95% CI 80.6%–95.9%). Individually, the experts had 85.7–89.8% accuracy and 89.2–92.3% F1 score. Three experts concurred on every patient in the test set; the fourth expert disagreed with their colleagues on 12% of the patients. The complete performance metrics of the majority expert opinion for both the training and test sets are shown in Table 1. There was no statistically significant difference between the majority expert opinion using all six canals and either the best-performing six-canal (*p* = 0.65) or two-canal machine learning models (*p* = 0.56).

### Classification by VOR gain cutoff

In the training set, a binary classifier using the optimal VOR gain cutoff of < 0.73 identified VN with 84.9% accuracy (95% CI 80.6%–94.9%) and 86.9% F1 score (95% CI 82.5%–91.0%). When the classifier applied the same cutoff value to the test set, it only achieved 75.5% accuracy (95% CI 63.3%–85.7%) and 82.4% F1 score (95% CI 70.8%–90.9%). This classifier’s performance was not statistically different from that of the best six-canal machine learning model (*p* = 0.10). However, the best-performing two-canal machine learning model (developed using the training set) achieved superior performance in the test set than the optimal VOR cutoff from the training set (*p* = 0.01). The full performance metrics of the classifier that used a VOR gain cutoff are shown in Table 1.

### Findings in subgroups of interest

The inferior subtype of VN is characterized on VHIT by isolated impairment of the ipsilateral posterior canal [29, 30]. In the training set, there were only two inferior VN patients (1% of all VN cases). One was incorrectly classified by the best two-canal and six-canal models as well as the VOR gain cutoff and blinded experts. The second was correctly classified by both machine learning models, but wrongly labeled as stroke by the gain cutoff and experts. In the test set, there were also two inferior VNs (6% of all VN). Both were incorrectly categorized as stroke by all classification methods (best two-canal and six-canal models, experts and VOR gain cut-off).

Strokes in the anterior inferior cerebellar artery (AICA) territory can have diverse results on VHIT [31]. There were no AICA strokes in the test set. In the training set, there were 7 AICA territory strokes which had variable VHIT patterns ranging from normal to bilateral abnormalities in several canals. None had a VHIT pattern typical for VN. The best six-canal model identified all seven cases correctly. The best two-canal model only misclassified one patient as VN (which had asymmetric impairment of both horizontal canals), while the blinded experts incorrectly categorized a different patient. These two cases were both mislabeled by the VOR gain cutoff.

## Discussion

We have developed machine learning models for classifying AVS patients as either VN or PCS using only unedited data from the VHIT and evaluated them on a test set collected at another institution. The performance of our machine learning models on this task was not significantly different from that of clinicians with neuro-otology expertise, and our findings imply that only the two horizontal canals need to be tested. We suggest that the VHIT combined with our models could be incorporated into the current AVS diagnostic workflow in the Emergency Room (Fig. 1b). For non-expert clinicians, this would replace HINTS plus and, as a horizontal canal VHIT can be performed at the bedside by any trained healthcare professional in 5 min, this would still allow for rapid clinical assessment of AVS patients. The VHIT results would be the input for our model, which would then indicate the likely diagnosis within seconds.

### Models versus VOR gain cut-off

The most commonly proposed method in the literature for automated classification methods using VHIT has been to use an optimal cut-off value of horizontal canal VOR gain to separate VN and PCS [13, 14, 32–35]. This method has achieved up to 90% accuracy; furthermore, compared to other VHIT metrics such as saccade velocity or prevalence, it is easier to use (as the gain is automatically calculated by the software and available at a glance) while demonstrating similar or superior results [34, 35]. However, one major issue with using a VOR gain cut-off is that the exact optimal value can vary greatly between populations. Although a value close to 0.70 has been suggested [36], the results in the literature have been achieved by cut-offs ranging from 0.57 to 0.93 [14, 15], which limits the widespread use of this method. Furthermore, the gain cut-off approach crudely reduces a set of head and eye traces into a single number, and thus may discount important information. For example, saccade features can identify VN even when gains are normal [18].

In contrast, our machine learning models considered the whole VHIT trace, encompassing not only the portion used to calculate the gain but also the section in which saccades appear. Rather than comparing the performance of our models in the test set against the optimal cut-off from the test set, it is more analogous to compare against the best cut-off from the training set; in this setting, our best machine learning model was superior to VOR gain cut-off, thereby demonstrating greater generalizability across clinical settings.

### Comparison of two-canal versus six-canal models

Limiting the training dataset for the machine learning models to only data from the two horizontal semicircular canals did not result in different performance from when all six canals were used; in fact, the best model overall used only two canals to classify patients with high accuracy and F1 score (87.8% and 90.9%). This suggests that using a truncated VHIT that only tests the horizontal canals may be sufficient, which would have benefits of being both shorter (5 min rather than 15) and easier technically [37]. However, as the six-canal models have to interpret three times the information, they require more training data and it is possible that a larger training dataset may unlock greater diagnostic capability.

Importantly, the six-canal models have the potential to be more useful for specific subgroups which can be diagnostically challenging. Inferior vestibular neuritis is a rare subtype of VN (~ 4% of all cases [30]) with isolated impairment of the inferior division of the vestibular nerve. It presents on bedside assessment with a down-beating torsional nystagmus and a negative bedside head impulse test (as the horizontal canals are unaffected) [29], i.e., a central HINTS. Furthermore, sparing of the horizontal canals means that it will also be misclassified by a VOR gain cut-off and potentially also by two-canal models. Both of the cases of inferior VN in the test set were misclassified by all methods, but in theory with enough training data, a six-canal model may learn that isolated impairment of one posterior canal is likely to be a form of VN. It is also possible that models which use the whole VHIT trace may be able to identify inferior VN using features beyond those currently used by clinicians. One case of inferior VN in the training set was correctly identified by not only the six-canal but also the two-canal model, despite horizontal canal traces which did not seem compatible with VN to experts. Extrapolation is limited by this single case and more data are needed.

AICA strokes can demonstrate bilateral, unilateral, or no impairment on bedside and video head impulse testing [31]. Unilateral impairment can result from damage to AICA-supplied structures such as the labyrinth, vestibular nucleus, root entry zone, and flocculus [38], and these cases can mimic a peripheral HINTS [39]. Similarly, the variability of their VHIT gain results means that they cannot be effectively identified using a VOR gain cut-off [40]. The 7 AICA cases in the training set (which did demonstrate diverse VHIT findings, although notably none had a typical VN pattern) were all correctly labeled by the six-canal model but not by the two-canal model, gain cut-off or blinded experts; this is a promising finding, but must be validated with more samples and using an external test set.

### Comparison with earlier studies

A previous study [15] also trained machine learning models on VHIT trace data to separate AVS patients into VN or PCS. They used data from the horizontal canals only, collected using a different VHIT system from 57 patients. Their neural network model’s accuracy of 87.9% was almost identical to that of our best model (87.8%). We used a larger dataset and an external test set for more robust results. Furthermore, their VHITs were reviewed for ‘data quality and artefacts’ by two neuro-otologists, and only ‘clean data with non-disruptive artefacts’ was included. In contrast, we tried to simulate the real-world setting of the intended clinical use (in which expert review after collection is unlikely to be available) by not performing post-testing quality control and achieved similar model performance. Their model’s accuracy was not significantly different from that of the optimal VOR gain cut-off (91.2%), which was a lower value (0.57) than ours; our use of an external test set allowed us to demonstrate the superiority of our models to a gain cut-off approach.

### Limitations

Only the VN patients in our training set who had atypical features received MRI, so it is possible that some were misdiagnosed. However, all the VN patients in the test set had MRI, so this would only impact the training process and not the validity of the evaluation. Other than VN and PCS, there are also less common causes of AVS such as vestibular migraine which can make up 10% of AVS [41] and which was not included in this study. Currently, stroke cannot be confidently excluded in a patient presenting with their first episode of vestibular migraine on the basis of history, bedside examination or VHIT (as it usually has normal findings [42]); nevertheless, future model iterations will include vestibular migraine patients with AVS in case machine learning models can help identify this group. The ICS impulse system we used is not the only commercially available VHIT system, but it is the most widely used in the literature [43]. Results from model evaluation using a single test set can be susceptible to variance. However, our results from the external test set were very similar to those obtained by combining the datasets and evaluating via 5-fold cross-validation, demonstrating robustness of our model’s results. Nevertheless, in future work, we will need to evaluate our model against data from multiple institutions to ensure the generalizability of our findings. We did not perform model calibration in this study due to the small test set size, but this will be a refinement step prior to clinical implementation once more external evaluation data are collected. Finally, we did not collect sociodemographic information beyond age and sex, and thus it was not possible to evaluate for relevant biases; this will also need to be addressed in the next iteration of the model.

## Summary

The present study demonstrated that machine learning models can effectively classify AVS patients as vestibular neuritis or posterior circulation stroke using only raw, unedited data from the VHIT. Our models showed generalizability across clinical settings when validated using an external test dataset. They are likely to be helpful as a diagnostic decision aid for Emergency Room clinicians without access to neuro-otology expertise in their assessment of AVS patients.

## Data and code availability

The study dataset contains patient health data and is not publicly available for privacy reasons. A deidentified version of the dataset is available on reasonable request from the corresponding author M.W. via a data sharing agreement. With this mediated access, the data are FAIR compliant. The code is similarly also available on reasonable request.

## Supplementary Information

Below is the link to the electronic supplementary material.

Supplementary file1 (PDF 172 KB)

Supplementary file2 (PDF 128 KB)

Supplementary file3 (PDF 147 KB)

Supplementary file4 (PDF 190 KB)

Supplementary file5 (PDF 186 KB)
