# Supplementary material for: Separation of stroke from vestibular neuritis using the video head impulse test: machine learning models versus expert clinicians
Source: J Neurol. 2025 Mar 5;272(3):248. doi: 10.1007/s00415-025-12918-3 (PMC11882619; doi:10.1007/s00415-025-12918-3)
Supplement: Supplementary file 1 — Supplementary file1 (PDF 172 KB) [file 415_2025_12918_MOESM1_ESM.pdf]

Article Title: Separation of Stroke from Vestibular Neuritis using the Video Head Impulse Test: Machine Learning Models versus Expert Clinicians

Authors: Chao Wang, Jeevan Sreerama, Benjamin Nham, Nicole Reid, Nese Ozalp, James O. Thomas, Cecilia Cappelen-Smith, Zeljka Calic, Andrew P. Bradshaw, Sally M. Rosengren, Deborah A. Black, Gülden Akdal, G. Michael Halmagyi, Mukesh Prasad, Gnana K. Bharathy, Miriam S. Welgampola

Journal: Journal of Neurology

Corresponding Author: Miriam S. Welgampola; Central Clinical School, University of Sydney, Australia; miriam@icn.usyd.edu.au

---

Supplemental Methods: VHIT Data Preparation for Machine Learning

Plain Language Summary

Firstly, the data was extracted as multiple Excel files from a database of VHIT results and merged into a single dataset, retaining only relevant columns. The data then underwent a filtering process based on maximum head velocity as a form of data standardisation. Additional filtering was applied to ensure that each patient had at least 10 valid test impulses in all 6 semicircular canals. Data from the first 10 valid impulses from each canal was then extracted for each patient. The data was split into training and test sets. Further processing included categorising variables, encoding of categorical variables, and standardising numerical data. Finally, the data was reshaped and transposed to suit LSTM (Long Short-Term Memory) model requirements for time series analysis.

Data Preparation and Filtering

- Data was extracted as CSV files from a Microsoft Access database of all the VHIT data, which was previously imported as XML files exported from the ICS Impulse VHIT software.
- The CSV files were read into a pandas DataFrame.
- Initial data exploration: checked data types, descriptive statistics, and missing values. Ensured that each Impulse ID had exactly 175 Timepoints and each timepoint had corresponding HeadVelocity and EyeVelocity data. Note that the dataset did not have any missing data.
- Identified the maximum HeadVelocity recorded for each Impulse ID. Applied filtering conditions based on MaxHeadVelocity and Plane (representing the testing plane, with 3 options of Lateral, LARP and RALP): excluded 'Lateral' plane impulses with MaxHeadVelocity > 250, and excluded 'LARP' or 'RALP' plane impulses with MaxHeadVelocity > 200
- Further data filtering: ensured that each Patient ID had at least 10 unique Impulse ID records per combination of Plane and Side (a binary variable indicating whether impulse was collected from the left or right side). Note that the combination of the Plane and Side variables determines which of the 6 semicircular canals the data was collected from.

- Impulse selection: applied the `retain_n_impulses` function to keep 10 impulses (Impulse ID) for each combination of Patient ID and Plane and Side
- Sorted the DataFrame based on Patient ID, Plane, Side, Impulse ID, and Timepoint.

Data Transformation

- Split the data into training and test sets based on Patient ID.
- Converted categorical columns to the `category` data type.
- Encoded categorical variables using `LabelEncoder`.
- Standardised numerical columns using `StandardScaler`.
- Reshaped the data for LSTM (Long Short-Term Memory) Models using function `reshape_data_for_lstm`, ensuring each sequence had 175 time points.
- Data transposed to fit the input format required by `sktime` models.
