# Supplementary material for: Separation of stroke from vestibular neuritis using the video head impulse test: machine learning models versus expert clinicians
Source: J Neurol. 2025 Mar 5;272(3):248. doi: 10.1007/s00415-025-12918-3 (PMC11882619; doi:10.1007/s00415-025-12918-3)
Supplement: Supplementary file 2 — Supplementary file2 (PDF 128 KB) [file 415_2025_12918_MOESM2_ESM.pdf]

Article Title: Separation of Stroke from Vestibular Neuritis using the Video Head Impulse Test: Machine Learning Models versus Expert Clinicians

Authors: Chao Wang, Jeevan Sreerama, Benjamin Nham, Nicole Reid, Nese Ozalp, James O. Thomas, Cecilia Cappelen-Smith, Zeljka Calic, Andrew P. Bradshaw, Sally M. Rosengren, Deborah A. Black, Gülden Akdal, G. Michael Halmagyi, Mukesh Prasad, Gnana K. Bharathy, Miriam S. Welgampola

Journal: Journal of Neurology

Corresponding Author: Miriam S. Welgampola; Central Clinical School, University of Sydney, Australia; miriam@icn.usyd.edu.au

Supplemental Table 1: Metadata of Variables Used for Machine Learning Model Development

Variable Name	Data Type	Categories (if categorical)/Units (if numerical)	Description
**Diagnosis**	Categorical (Nominal)	Vestibular Neuritis/Posterior Circulation Stroke	Patient's diagnosis, either vestibular neuritis or posterior circulation stroke
**Patient ID**	Categorical (Nominal)	Unique identifier for each patient	Patient identifier
**Plane**	Categorical (Nominal)	Horizontal/RALP (Right Anterior, Left Posterior)/LARP (Left Anterior, Right Posterior)	Which plane the data was collected in (VHIT testing is sequentially performed in 3 planes, each with 2 semicircular canals) (linked to Patient ID)
**Side**	Categorical (Nominal)	Left/Right	Which side's semicircular canals the data was collected from (linked to Plane); note that the combination of plane and side values identifies which of the 6 semicircular canals the data was collected from (Canal ID from Figure 2)
**Impulse ID**	Categorical (Nominal)	Identifier for each impulse	Identifier for each individual head impulse performed during VHIT (linked to Side)
**Timepoint**	Numerical (Integer)	Units (each represents 4 ms)	Represents the 175 individual timepoints across the 750 ms duration of the head impulse (linked to Impulse ID)
**Head Velocity**	Numerical	°/s	Head velocity recorded at associated timepoint (linked to Timepoint)
**Eye Velocity**	Numerical	°/s	Eye velocity recorded at associated timepoint (linked to Timepoint)

VHIT = video head impulse test
