# Supplementary material for: Separation of stroke from vestibular neuritis using the video head impulse test: machine learning models versus expert clinicians
Source: J Neurol. 2025 Mar 5;272(3):248. doi: 10.1007/s00415-025-12918-3 (PMC11882619; doi:10.1007/s00415-025-12918-3)
Supplement: Supplementary file 3 — Supplementary file3 (PDF 147 KB) [file 415_2025_12918_MOESM3_ESM.pdf]

**Article Title: Separation of Stroke from Vestibular Neuritis using the Video Head Impulse Test: Machine Learning Models versus Expert Clinicians**

**Authors**: Chao Wang, Jeevan Sreerama, Benjamin Nham, Nicole Reid, Nese Ozalp, James O. Thomas, Cecilia Cappelen-Smith, Zeljka Calic, Andrew P. Bradshaw, Sally M. Rosengren, Deborah A. Black, Gülden Akdal, G. Michael Halmagyi, Mukesh Prasad, Gnana K. Bharathy, Miriam S. Welgampola

**Journal:** Journal of Neurology

**Corresponding Author:** Miriam S. Welgampola; Central Clinical School, University of Sydney, Australia; miriam@icn.usyd.edu.au

## Supplemental Table 2: Patient Characteristics

| Characteristic | First Cohort (Training Set) (n = 252) | | Second Cohort (Test Set) (n = 49) | | Statistical Testing |
|---|---|---|---|---|---|
| | Vestibular Neuritis | Posterior Circulation Stroke | Vestibular Neuritis | Posterior Circulation Stroke | |
| **n (%)** | 149 (59) | 103 (41) | 33 (67) | 16 (33) | N/A |
| **Sex, female, n (%)** | 57 (38) | 30 (29) | 11 (33) | 6 (38) | $X^2(1) = 0.28, p = 0.60$ [a]<br>$X^2(1) = 0.46, p = 0.50$ [b] |
| **Age, years, mean (SD)** | 56.2 (17.1) | 66.7 (11.2) | 61.3 (13.1) | 68.1 (11.8) | $U = 2145, p = 0.25$ [a]<br>$t(117) = 0.45, p = 0.66$ [b] |

[a] Comparison between vestibular neuritis patients of first cohort and second cohort

[b] Comparison between posterior circulation stroke patients of first cohort and second cohort
