# Supplementary material for: Separation of stroke from vestibular neuritis using the video head impulse test: machine learning models versus expert clinicians
Source: J Neurol. 2025 Mar 5;272(3):248. doi: 10.1007/s00415-025-12918-3 (PMC11882619; doi:10.1007/s00415-025-12918-3)
Supplement: Supplementary file 4 — Supplementary file4 (PDF 190 KB) [file 415_2025_12918_MOESM4_ESM.pdf]

Article Title: Separation of Stroke from Vestibular Neuritis using the Video Head Impulse Test: Machine Learning Models versus Expert Clinicians

Authors: Chao Wang, Jeevan Sreerama, Benjamin Nham, Nicole Reid, Nese Ozalp, James O. Thomas, Cecilia Cappelen-Smith, Zeljka Calic, Andrew P. Bradshaw, Sally M. Rosengren, Deborah A. Black, Gülden Akdal, G. Michael Halmagyi, Mukesh Prasad, Gnana K. Bharathy, Miriam S. Welgampola

Journal: Journal of Neurology

Corresponding Author: Miriam S. Welgampola; Central Clinical School, University of Sydney, Australia; miriam@icn.usyd.edu.au

Supplemental Table 3: Performance metrics of all machine learning classification models for separating posterior circulation stroke and vestibular neuritis

	Accuracy, %	Precision, %	Sensitivity, %	F1-score, %	Specificity, %	AUC
**Training Set**						
**All Canal Models**						
**Rocket**	92.5 (88.9-92.5)	94.5 (90.9-97.9)	92.6 (87.9-96.6)	93.6 (90.3-96.2)	92.2 (86.4-97.1)	0.92 (0.89-0.96)
**Arsenal**	91.7 (88.5-94.8)	92.7 (89.0-96.5)	93.3 (89.3-97.3)	93.0 (89.7-95.7)	89.3 (83.5-95.2)	0.96 (0.94-0.99)
**RIC**	100 (100-100)	100 (100-100)	100 (100-100)	100 (100-100)	100 (100-100)	1.00 (1.00-1.00)
**catch22**	99.6 (98.8-100)	100 (100-100)	99.3 (98.0-100)	99.7 (98.9-100)	100 (100-100)	1.00 (1.00-1.00)
**Horizontal Canal Models**						
**Rocket**	92.9 (89.7-95.6)	95.8 (92.6-98.6)	91.9 (87.3-96.0)	93.8 (90.9-96.5)	94.2 (89.3-98.1)	0.93 (0.90-0.96)
**Arsenal**	88.9 (84.9-92.5)	91.2 (86.9-95.2)	89.9 (85.2-94.6)	90.5 (86.9-93.8)	87.4 (80.6-93.2)	0.93 (0.90-0.97)
**RIC**	100 (100-100)	100 (100-100)	100 (100-100)	100 (100-100)	100 (100-100)	1.00 (1.00-1.00)
**catch22**	99.2 (98.0-100)	100 (100-100)	98.7 (96.6-100)	99.3 (98.3-100)	100 (100-100)	1.00 (1.00-1.00)
**Test Set**						
**All Canal Models**						
**Rocket**	83.7 (73.5-93.9)	87.9 (79.5-96.8)	87.9 (75.8-97.0)	87.9 (78.0-95.5)	75.0 (56.3-93.8)	0.81 (0.69-0.94)
**Arsenal**	81.6 (71.4-91.8)	85.3 (76.3-94.1)	87.9 (75.8-97.0)	86.6 (76.4-94.3)	68.8 (43.8-87.5)	0.78 (0.62-0.95)
**RIC**	77.6 (65.3-87.8)	84.4 (75.0-93.8)	81.8 (66.7-93.9)	83.1 (72.7-91.8)	68.8 (43.8-87.5)	0.81 (0.67-0.96)
**catch22**	71.4 (59.2-83.7)	80.6 (70.8-91.7)	75.8 (60.6-90.9)	78.1 (65.6-88.6)	62.5 (37.5-87.5)	0.69 (0.53-0.85)
**Horizontal Canal Models**						
**Rocket**	87.8 (77.6-95.9)	90.9 (82.9-100)	90.9 (81.8-100)	90.9 (82.8-97.1)	81.3 (62.5-100)	0.86 (0.75-0.97)
**Arsenal**	85.7 (75.5-93.9)	88.2 (80.0-96.9)	90.9 (78.8-100)	89.6 (80.6-95.9)	75.0 (50.0-93.8)	0.88 (0.77-0.99)
**RIC**	81.6 (69.4-91.8)	85.3 (76.5-93.9)	87.9 (75.8-97.0)	86.6 (76.9-94.3)	68.8 (43.8-87.5)	0.84 (0.71-0.96)
**catch22**	81.6 (71.4-91.8)	85.3 (76.3-93.9)	87.9 (75.8-97.0)	86.6 (76.5-94.4)	68.8 (43.8-87.5)	0.87 (0.78-0.97)

Models are named for the algorithm used for development. Data from all 6 semicircular canals was used to train the “All Canal” Models, whereas the Horizontal Canal Models only used data from the 2 horizontal canals. Vestibular neuritis was defined as the positive class. Models using Rocket output a binary classification. Models using the other algorithms output a predicted probability, which was

converted into binary class labels using a cut-off of 0.5. The brackets indicate 95% CI. F1-score is the harmonic mean of precision and recall (sensitivity). AUC = area under the receiver operating characteristic curve; RIC = random interval classifier
