# Supplementary material for: Separation of stroke from vestibular neuritis using the video head impulse test: machine learning models versus expert clinicians
Source: J Neurol. 2025 Mar 5;272(3):248. doi: 10.1007/s00415-025-12918-3 (PMC11882619; doi:10.1007/s00415-025-12918-3)
Supplement: Supplementary file 5 — Supplementary file5 (PDF 186 KB) [file 415_2025_12918_MOESM5_ESM.pdf]

**Article Title: Separation of Stroke from Vestibular Neuritis using the Video Head Impulse Test: Machine Learning Models versus Expert Clinicians**

**Authors**: Chao Wang, Jeevan Sreerama, Benjamin Nham, Nicole Reid, Nese Ozalp, James O. Thomas, Cecilia Cappelen-Smith, Zeljka Calic, Andrew P. Bradshaw, Sally M. Rosengren, Deborah A. Black, Gülden Akdal, G. Michael Halmagyi, Mukesh Prasad, Gnana K. Bharathy, Miriam S. Welgampola

**Journal:** Journal of Neurology

**Corresponding Author:** Miriam S. Welgampola; Central Clinical School, University of Sydney, Australia; miriam@icn.usyd.edu.au

## Supplemental Table 4: Performance metrics of machine learning classification models for separating posterior circulation stroke and vestibular neuritis when developed using pooled patients from both institutions with an 80/20 training/test split

| | Accuracy, % | Precision, % | Sensitivity, % | F1-score, % | Specificity, % |
|---|---|---|---|---|---|
| **Training Set** | | | | | |
| **All Canal Models** | | | | | |
| **Rocket** | 97.7 (95.5-99.8) | 98.2 (96.5-99.9) | 97.9 (95.9-100) | 98.1 (96.3-99.9) | 97.3 (94.8-99.7) |
| **Arsenal** | 91.2 (89.5-92.9) | 92.5 (90.8-94.2) | 93.0 (91.7-94.3) | 92.7 (91.4-94.1) | 88.4 (85.7-91.1) |
| **RIC** | 100 (100-100) | 100 (100-100) | 100 (100-100) | 100 (100-100) | 100 (100-100) |
| **catch22** | 99.0 (98.5-99.5) | 99.3 (98.9-99.7) | 99.0 (98.4-99.7) | 99.2 (98.8-99.6) | 98.9 (98.3-99.6) |
| **Horizontal Canal Models** | | | | | |
| **Rocket** | 91.8 (90.8-92.8) | 93.8 (92.7-94.8) | 92.6 (91.0-94.1) | 93.2 (92.3-94.0) | 90.5 (88.8-92.3) |
| **Arsenal** | 88.4 (87.0-89.7) | 90.9 (89.3-92.5) | 89.8 (88.1-91.6) | 90.3 (89.2-91.4) | 86.1 (83.4-88.9) |
| **RIC** | 100 (100-100) | 100 (100-100) | 100 (100-100) | 100 (100-100) | 100 (100-100) |
| **catch22** | 99.4 (99.2-99.6) | 99.9 (99.6-100) | 99.2 (98.7-99.7) | 99.5 (99.3-99.7) | 99.8 (99.4-100) |
| **Test Set** | | | | | |
| **All Canal Models** | | | | | |
| **Rocket** | 84.7 (79.3-90.1) | 87.0 (82.9-91.0) | 87.9 (80.2-95.5) | 87.3 (82.3-92.2) | 79.9 (73.5-86.3) |
| **Arsenal** | 81.7 (77.9-85.5) | 85.0 (79.9-90.2) | 85.2 (79.8-90.5) | 84.9 (81.7-88.1) | 76.6 (67.2-86.0) |
| **RIC** | 83.4 (79.1-87.7) | 85.0 (79.9-90.1) | 88.5 (83.5-93.4) | 86.5 (83.1-90.0) | 75.7 (66.3-85.0) |
| **catch22** | 78.4 (72.6-84.2) | 83.4 (76.2-90.6) | 81.3 (72.0-90.5) | 81.8 (76.5-87.1) | 73.9 (60.8-87.0) |
| **Horizontal Canal Models** | | | | | |
| **Rocket** | 82.4 (78.4-86.4) | 86.6 (80.3-93.0) | 84.7 (78.2-91.1) | 85.3 (82.0-88.6) | 79.1 (67.9-90.2) |
| **Arsenal** | 82.7 (77.5-87.9) | 86.6 (79.7-93.6) | 85.2 (80.1-90.2) | 85.7 (81.6-89.7) | 79.1 (67.1-91.1) |
| **RIC** | 83.0 (79.0-87.1) | 85.8 (78.9-92.7) | 87.4 (81.7-93.0) | 86.2 (83.2-89.3) | 76.5 (64.0-89.0) |
| **catch22** | 79.1 (74.3-83.9) | 84.0 (78.7-89.3) | 81.3 (73.4-89.3) | 82.3 (77.8-86.9) | 75.7 (65.7-85.7) |

Models are named for the algorithm used for development. Data from all 6 semicircular canals was used to train the "All Canal" Models, whereas the Horizontal Canal Models only used data from the 2 horizontal canals. Vestibular neuritis was defined as the positive class. The brackets indicate 95% CI. F1-score is the harmonic mean of precision and recall (sensitivity). RIC = random interval classifier
